# Quantitative succinyl-proteome profiling of Chinese hickory (*Carya cathayensis*) during the grafting process

**DOI:** 10.1186/s12870-019-2072-8

**Published:** 2019-11-04

**Authors:** Huwei Yuan, Juanjuan Chen, Ying Yang, Chenjia Shen, Dongbin Xu, Junfeng Wang, Daoliang Yan, Yi He, Bingsong Zheng

**Affiliations:** 1 State Key Laboratory of Subtropical Silviculture, Zhejiang A&F University, Hangzhou, 311300 People’s Republic of China; 2 Center for Cultivation of Subtropical Forest Resources (CCSFR, Zhejiang A&F University, Hangzhou, 311300 People’s Republic of China; 3 College of Life and Environmental Sciences Hangzhou Normal University, Hangzhou, 310036 People’s Republic of China

**Keywords:** Chinese hickory, Post-translational modification, Succinylation, Grafting, Heat shock proteins

## Abstract

**Background:**

Chinese hickory (*Carya cathayensis*) is a popular nut plant having high economic value. Grafting is applied to accelerate the transition from vegetative phase to reproductive phase. Lysine succinylation occurs frequently in the proteins associated with metabolic pathways, which may participate in the regulation of the grafting process. However, the exact regulatory mechanism underlying grafting process in Chinese hickory has not been studied at post-translational modification level.

**Results:**

A comprehensive proteome-wide lysine succinylation profiling of Chinese hickory was explored by a newly developed method combining affinity enrichment and high-resolution LC-MS/MS. In total, 259 succinylation sites in 202 proteins were identified, representing the first comprehensive lysine succinylome in Chinese hickory. The succinylation was biased to occur in the cytosolic proteins of Chinese hickory. Moreover, four conserved succinylation motifs were identified in the succinylated peptides. Comparison of two grafting stages of Chinese hickory revealed that the differential expressed succinylated proteins were mainly involved in sugar metabolism, carbon fixation, amino acid metabolism and plant-pathogen interaction. Besides, seven heat shock proteins (HSPs) with 11 succinylation sites were also identified, all of which were observed to be up-regulated during the grafting process.

**Conclusions:**

Succinylation of the proteins involved in amino acid biosynthesis might be required for a successful grafting. Succinylated HSPs might play a role in stress tolerance of the grafted Chinese hickory plants. Our results can be a good resource for functional validation of the succinylated proteins and a starting point for the investigation of molecular mechanisms during lysine succinylation occurring at grafting site.

## Background

Chinese hickory (*Carya cathayensis* Sarg.) is an economically important plant in China which produces nuts with considerable amounts of nutritious components [1]. A mature Chinese hickory nut usually contains not only an extremely high proportion of unsaturated fatty acids (over 70%), including oleic acid, palmitic and linolenic acid, but also several antitumor compounds, like phenolics, carayensin-A, carayensin-B and carayensin-C [2–4]. Limited to its long juvenile phase, the breeding efficiency and yield of Chinese hickory are largely reduced, which has limited the long-term development of Chinese hickory horticultural industry [5].

Grafting is an ancient technique that can provide an effective way to accelerate the transition from the vegetative phase to reproductive phase in Chinese hickory [6]. A successful grafting process is controlled by both intrinsic hereditary determinants and external environmental factors [7, 8]. Recent studies have revealed the response of important genes and proteins involved in the grafting process of Chinese hickory. For example, 12 cambium formation and cell growth-related genes were up-regulated during the grafting process [6]. Transcriptomic analysis of Chinese hickory uncovered a great number of auxin- and cytokinin-related differentially expressed genes, suggesting a role of hormone signaling pathways during the graft process of Chinese hickory [5]. Proteomic analysis of the graft unions in Chinese hickory showed that five flavonoid biosynthesis-related proteins, including flavanone-3-hyfroxylase, cinnamate-4-hydroxylase, dihydroflavonol-4-reductase, chalcone synthase and chalcone isomerase, were significantly up-regulated after grafting, indicating the involvement of secondary metabolism during the grafting process [9]. Recently, a grafting response gene, *CcPIP1;2*, was cloned and functionally characterized. Over-expression of *CcPIP1;2* in *Arabidopsis* could increase the resistance to abiotic stresses by regulating the expression of ABA-related genes as well as cell wall expansion genes [8]. However, the molecular mechanism underlying the post-translational modifications (PTMs) of grafting-induced proteins is largely unknown.

PTM of proteins is an efficient biological mechanism controlling many biological processes, such as transcription, metabolism, and aging [10]. In both eukaryotic and prokaryotic cells, PTMs expand the functional and structural diversity of a limited number of proteins [11]. In the past few decades, many studies have focused on reversible acetylation at lysine residues [12]. In addition to acetylation, lysine residues can be modified by various short-chain acylations, such as propionylation, butyrylation, crotonylation, malonylation, succinylation and glutarylation [12–14]. Lysine succinylation has been studied in a number of organisms, including microbes (such as *Mycobacterium tuberculosis* and *Saccharomyces cerevisiae*), mammals (such as *Rattus norvegicus* and *Mus musculus*), and plants (*Taxus × media* and *Dendrobium officinale*) [15–18].

Lysine succinylation occurs frequently in the proteins associated with metabolic pathways, which may participate in the regulation of the grafting process [19, 20]. Thus, identifying succinylated proteins may provide useful information on how PTMs are involved in the grafting process of Chinese hickory. Previous studies focused on the physiological and transcriptional aspects in Chinese hickory during the grafting process [1, 2, 21–27]. However, in best of our knowledge, no report on PTMs are available till date in Chinese hickory. Here, we report the first proteomics-based quantification of lysine succinylation in Chinese hickory graft union during the grafting process. Current study provides new insight into the molecular mechanism underlying the grafting process at the PTM level.

## Results

### Proteome-wide analysis of lysine succinylation sites of Chinese hickory

**Fig. 1:**
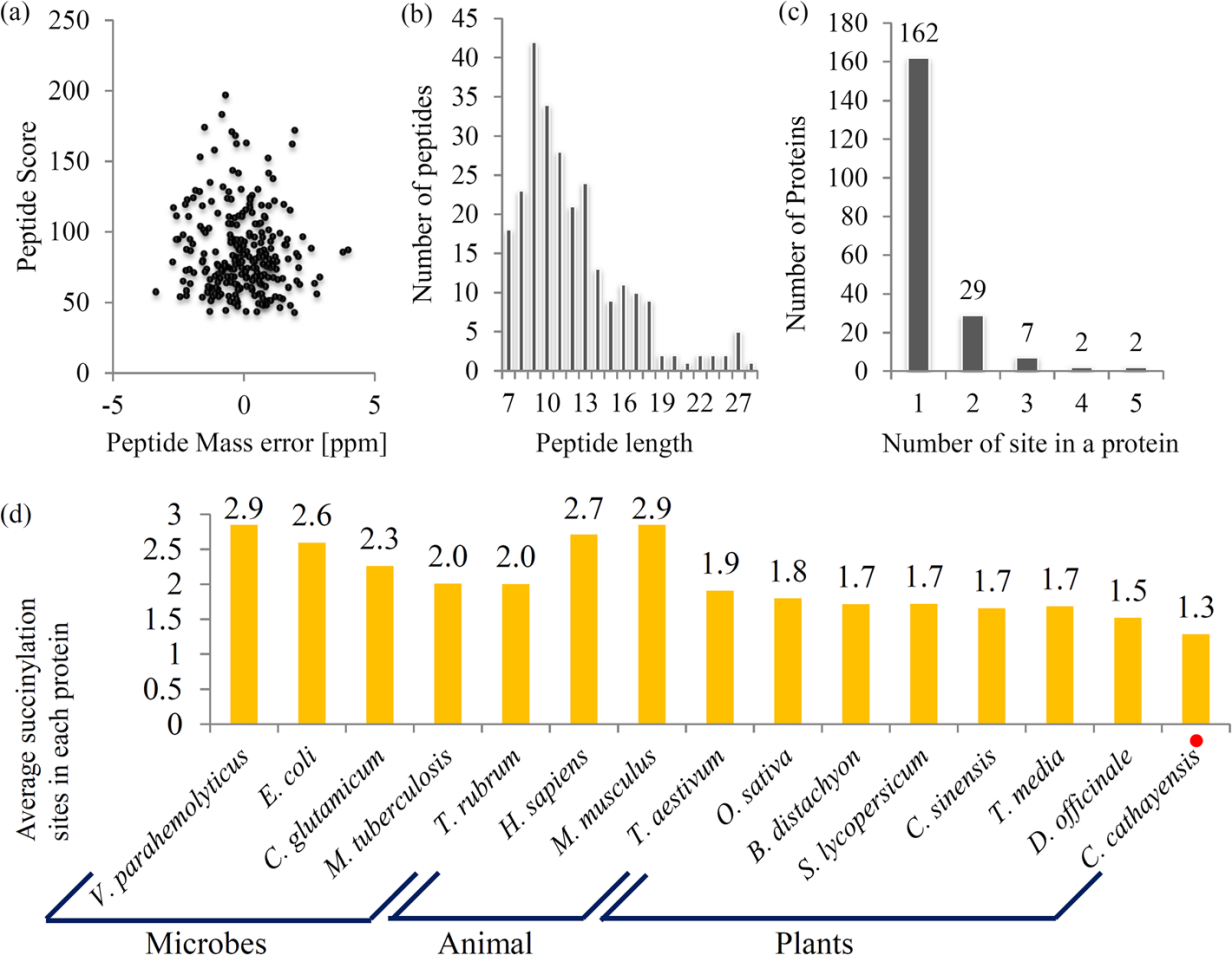
The basic information of LC-MS/MS data. **a** The peptide score of LC-MS/MS data. **b** Length distribution of succinylated peptides based on their length. **c** The number of proteins with different number of succinylated sites. **d** Average succinylation sites on each succinylated protein from various organisms

Lysine succinylation is a widespread PTM in both prokaryotic and eukaryotic cells, however, the succinylome of woody plants has not been reported previously [16]. A flow chart of the succinyl proteomics analysis has been provided (Additional file 1). Two important parameters, mass error and peptide length, were calculated. The mass errors of the most succinylated peptides were under 0.02 Da and the lengths of most succinylated peptides varied from 7 to 27 amino-acid residues (Fig. 1a and b).

Altogether, 259 succinylation sites in 202 proteins were identified, out of which 177 succinylation sites in 143 proteins were quantified (Additional file 2). Most of the succinylated proteins (162 proteins) contained only one succinylation site. Twenty nine succinylated proteins contained two succinylation sites, and 11 succinylated proteins contained more than three succinylation sites (Fig. 1c). Interestingly, two proteins, including phosphoglycerate kinase (CS_87744_c0) and protein disulfide-isomerase (CS_93322_c0), contained five succinylation sites. To analyze the number of succinylated proteins and the density of succinylation sites per protein, the succinylome of Chinese hickory was compared with those published in other species. The number of succinylated proteins in Chinese hickory was recorded significantly less than those in microbes and animals, including *E. coli* (990 proteins), *V. parahemolyticus* (750 proteins), *M. tuberculosis* (686 proteins), *H. sapiens* (738 proteins) and *M. musculus* (750 proteins), but almost similar to those in plants, such as *T. aestivum* (173 proteins), *S. lycopersicum* (202 proteins), *T. media* (193 proteins), *D. officinale* (207 proteins) [17, 18, 28–30] (Additional file 3). Meanwhile, it is observed that the average number of succinylation sites per protein in Chinese hickory was less than that in most reported species (Fig. 1d).

### Characterization of the Chinese hickory lysine succinylome

**Fig. 2:**
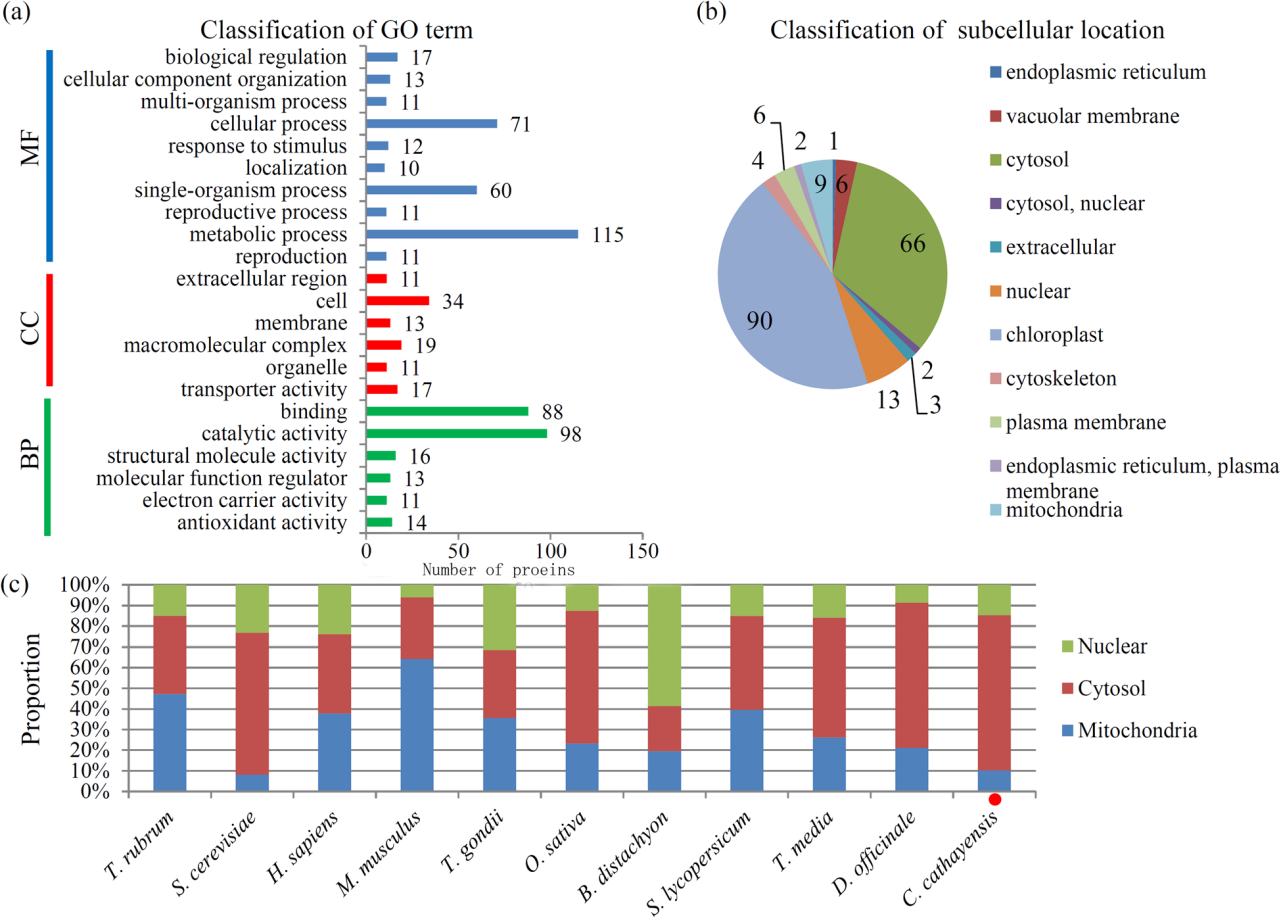
Bioinformatic analysis of lysine succinylation sites and succinylated proteins in Chinese hickory**. a** GO classifications for lysine succinylated proteins in Chinese hickory. **b** Subcellular locations of lysine succinylated proteins in Chinese hickory. **c** The proportions of succinylated proteins in mitochondria, cytoplasm and nucleus in different organisms

To predict the possible functions, all of identified succinylated proteins were classified into different GO categories. In ‘biological process’ category, most of the succinylated proteins were classified into the ‘binding’ (88 proteins) and ‘catalytic activity’ (98 proteins) terms; in ‘cellular component’ category, the largest group of succinylated proteins belonged to the ‘cell’ (34 proteins) and ‘macromolecular complex’ (19 proteins) terms; and in ‘molecular function’ category, the main terms were ‘metabolic process’ (115 proteins) and ‘cellular process’ (71 proteins) (Fig. 2a).

There is a close relativity between protein function and subcellular localization [30, 31]. With regard to the subcellular locations of all the identified succinylated proteins in Chinese hickory, the largest group was chloroplast-located proteins, followed by cytosol-located, nuclear-located, and mitochondria-located proteins, the number of which accounted for 44.6, 32.7, 6.4 and 4.5% for all of the identified succinylated proteins, respectively (Fig. 2b). Additionally, the relative proportions of succinylated proteins in the organelles like nuclei, cytosol and mitochondria in *C. cathayensis* were compared with other published organisms, including *T. rubrum*, *S. cerevisiae*, *H. sapiens*, *M. musculus*, *T. gondii*, *O. sativa*, *B. distachyon*, *S. lycopersicum*, *T. media* and *D. officinale* [16–18, 30–32]. Among these organisms, Chinese hickory possessed the highest proportion of cytosol-located succinylated proteins (75.0%), but the proportion of mitochondria-located succinylated proteins was only 10.2%, lower than that of all organisms mentioned above, excepting *S. cerevisiae* [16] (Fig. 2c).

### Motif analysis of identified lysine-succinylated peptides

**Fig. 3:**
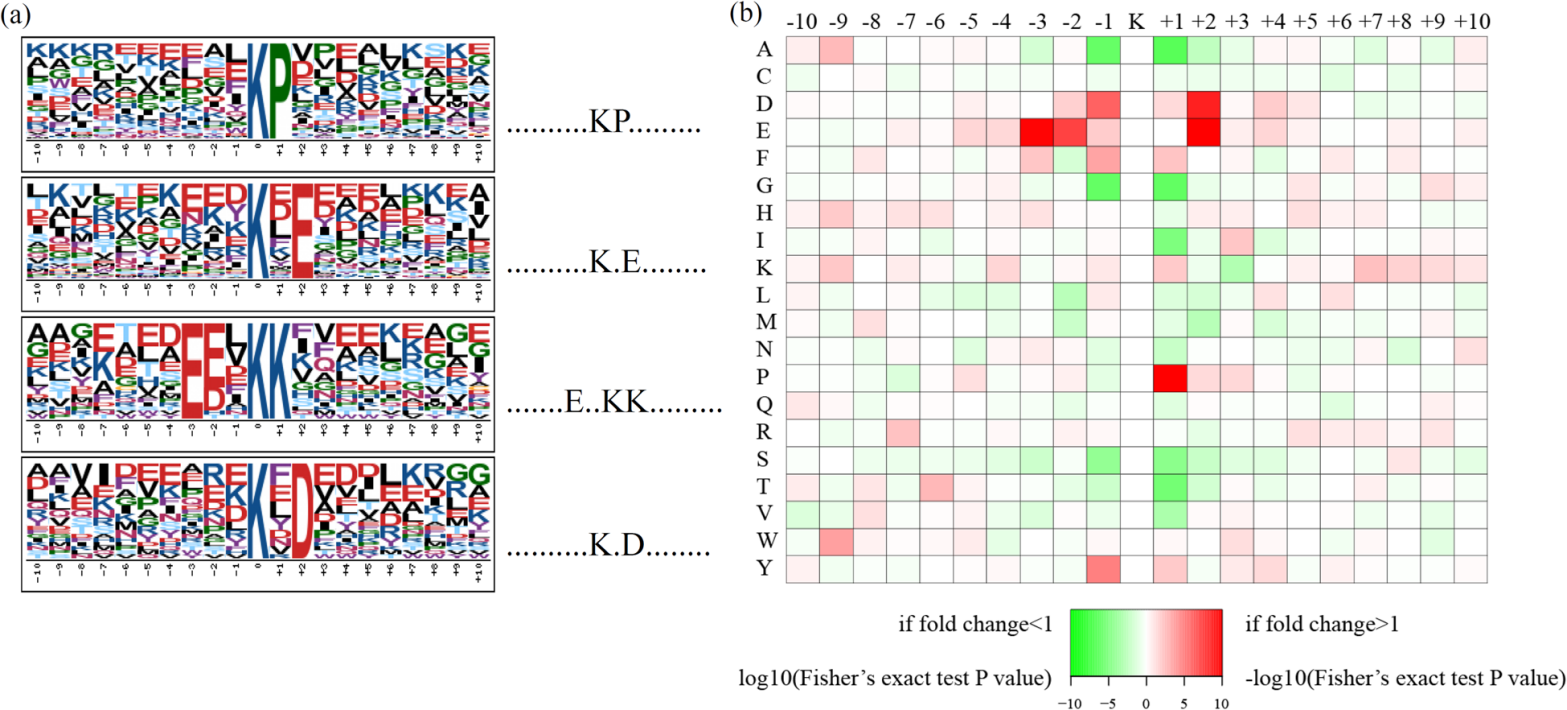
Plot shows relative abundance of amino acids flanking succinylated lysine**. a** Probability sequence motifs of succinylation sites consisting of 10 residues surrounding the targeted lysine residue using Motif-X. Four significantly enriched succinylation site motifs were identified. **b** The relative abundance was counted and schematically represented by an intensity map. The intensity map shows enrichment of amino acids in specific positions of succinylated lysine (10 amino acids upstream and downstream of the succinylation sites)

The patterns of amino acids surrounding succinylation sites are diverse among organisms but regular in a given organism [16, 18]. In the current study, the amino acid sequences of all succinylated peptides were extracted to analyze the typical motifs in the identified succinylated proteins of Chinese hickory. Several representative amino acids, such as proline (P), glutamic acid (E), lysine (K) and aspartic acid (D), were enriched downstream of the succinylated-lysine sites. In total, four preferred sequence patterns, including K^suc^P, K^suc^**·**E, E**··**K^suc^K, and K^suc^**·**D, were identified using software Motif-X with *P* at 0.000001 (Fig. 3). After comparison of the four motifs identified in Chinese hickory with those of the previously published succinylomes, it was found that these motifs were not unique but shared by both of *C. cathayensis* and *Camellia sinensis* [33].

### Comparison of succinylation sites on the enzymes related with the glycolysis and TCA cycle among various species

**Fig. 4:**
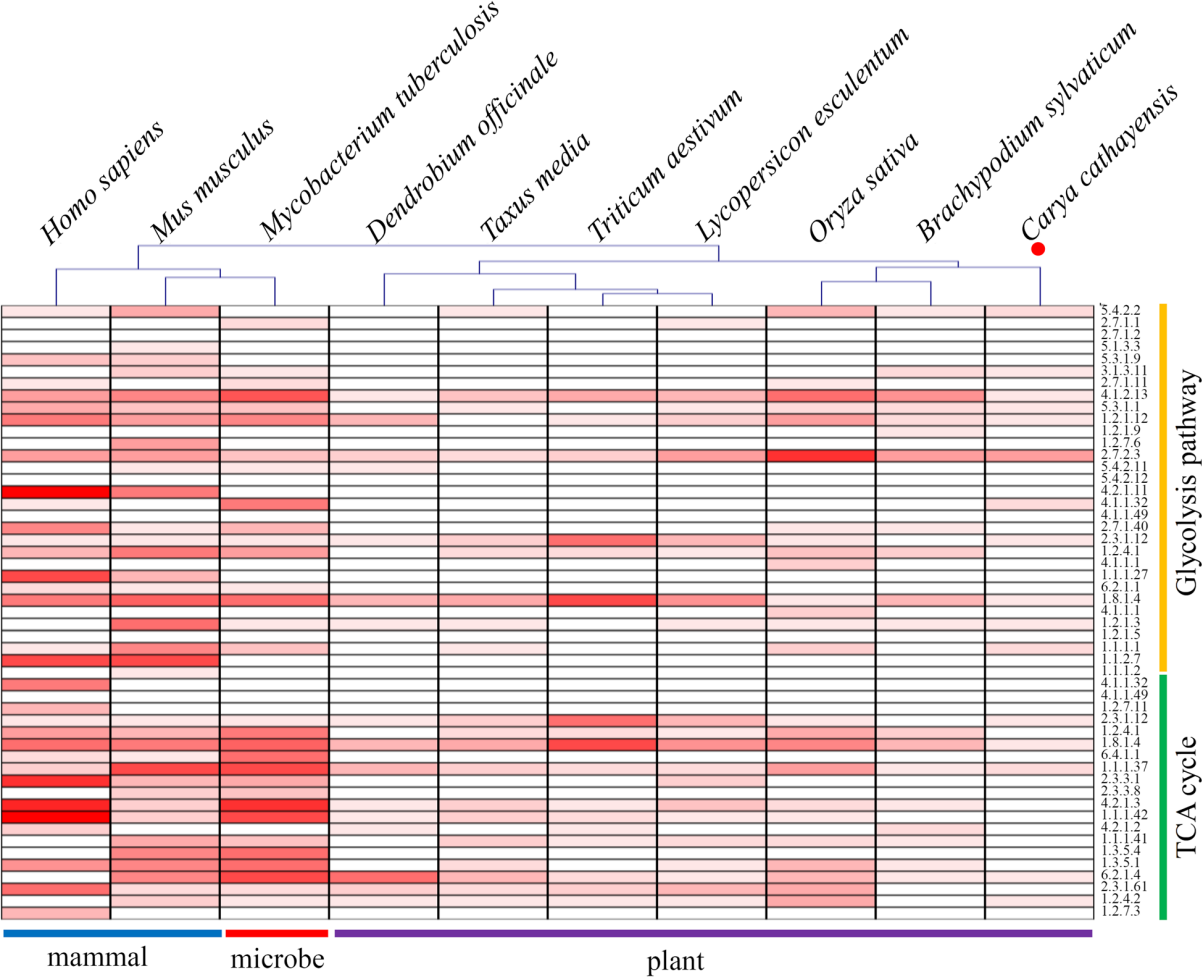
Comparison of succinylation sites in the enzymes involved in glycolysis and TCA cycle**.** A heatmap showed the numbers of succinylation sites in key enzymes involved in glycolysis and TCA cycle from various organisms. Red indicates succinylation site of the corresponding enzyme. Various shades of red indicates the differences in the number of succinylation sites. Deeper red represents more succinylation sites

In Chinese hickory, 11 key glycolytic enzymes, including phosphoglucomutase (PGM, EC: 5.4.2.2), fructose-1,6-bisphosphatase (FBP, EC: 3.1.3.11), fructose-bisphosphate aldolase (ALDO, EC: 4.1.2.13), triosephosphate isomerase (TPI, EC: 5.3.1.1), glyceraldehyde-3-phosphate dehydrogenase (GAPDH, EC: 1.2.1.12), phosphoglycerate kinase (PGK, EC: 2.7.2.3), phosphoeno/lpyruvate carboxykinase (GTP, EC: 4.1.1.32), dihydrolipoyllysine-residue acetyltransferase (DLAT, EC: 2.3.1.12), dihydrolipoyl dehydrogenase (DLD, EC: 1.8.1.4), aldehyde dehydrogenase (ALDH, EC: 1.2.1.3) and alcohol dehydrogenase (ADH, EC: 1.1.1.1), were identified as succinylated proteins (Fig. 4 and Additional file 4). Among these enzymes, PGK contained the largest number of succinylation sites (eight sites) and four enzymes, including FBP, GAPDH, DLAT and DLD contained only one succinylation site. For the TCA cycle, five key enzymes, including dihydrolipoyllysine-residue acetyltransferase (DLAT, EC: 2.3.1.12), dihydrolipoyl dehydrogenase (DLD, EC: 1.8.1.4), malate dehydrogenase (MDH, EC: 1.1.1.37), succinate-CoA ligase (SUCLG, EC: 6.2.1.4) and oxoglutarate dehydrogenase (OGDH, EC: 1.2.4.2) were observed to be lysine-succinylated (Fig. 4 and Additional file 5).

### Differences in lysine succinylation during the grafting process of Chinese hickory

In order to investigate the differences in lysine succinylation during the grafting process, a quantitative succinylome profiling was done in the current study (Additional file 6). A total of 68 differentially expressed succinylated proteins (DESPs) were identified, including 45 up-regulated DESPs with 54 succinylation sites and 23 down-regulated DESPs with 29 succinylation sites, at 7 d after grafting as compared with the control (0 d after grafting) (Additional file 7). Among these DESPs, cytosolic glutamine synthetase β2 (CS_88158_c0) and calcyclin-binding protein (CS_48795_c0) were up-regulated over 2 folds, but only one unknown protein was down-regulated over 2 folds.

### Functional enrichment-based clustering for quantitative category

**Fig. 5:**
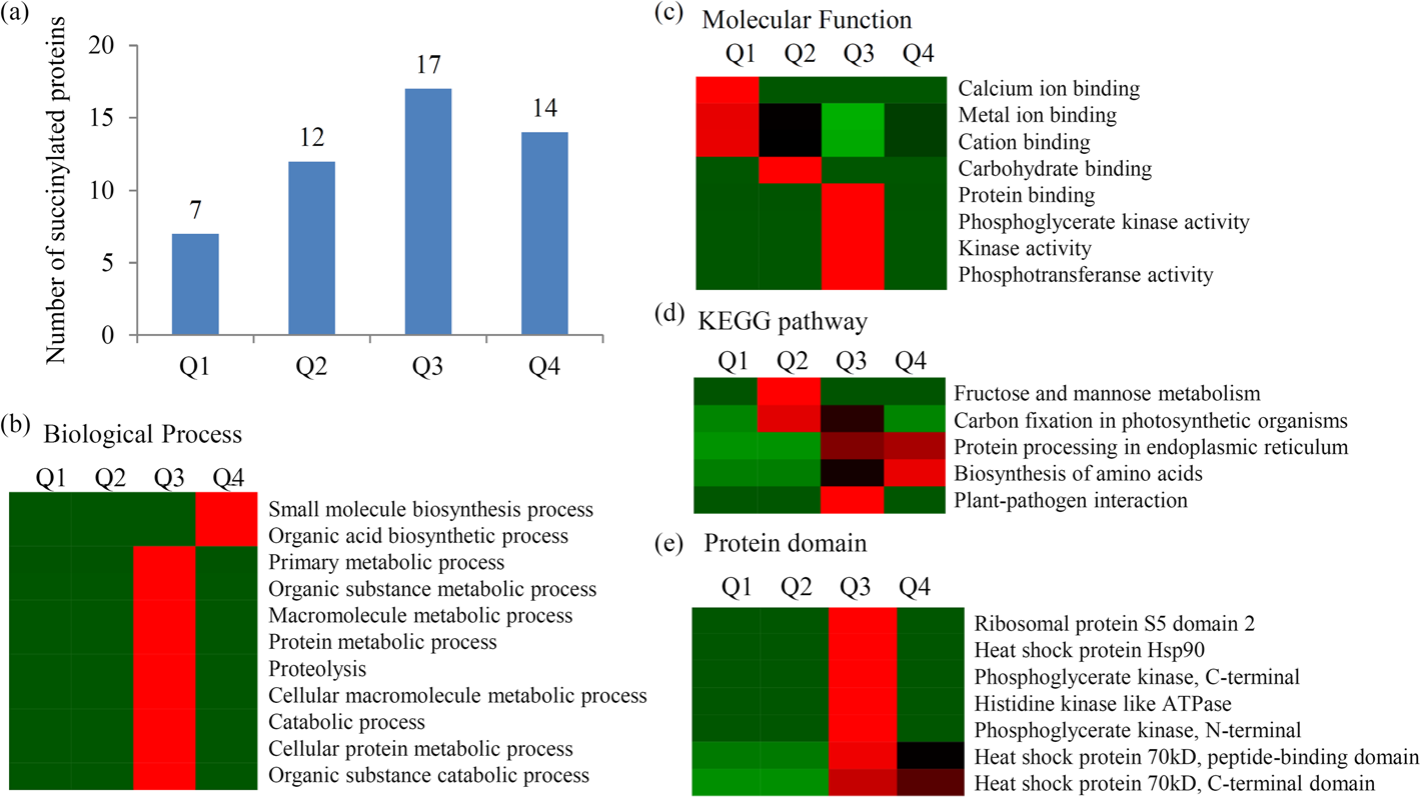
Differences in lysine succinylation during the grafting process of Chinese hickory**. a** Number of DESPs during the grafting process of Chinese hickory. Enrichment analysis of DESPs in Chinese hickory during grafting process. Significantly enriched GO terms of DESPs concerning biological process (**b**) and molecular function (**c**). **d** Significantly enriched KEGG terms of DESPs. **e** Significantly enriched protein domains of DESPs. For hierarchical clustering, protein categories that were enriched in at least one of the clusters with a *P*-value < 0.05 were transformed by the x = −log10 (*P*-value) function. These x-values were z-transformed and clustered by one-way hierarchical clustering basing on the Euclidean distance. The quantifiable proteins in this study were divided into four quantitative categories according to their D7/D0 Ratio: Q1 (0 < D7/D0 Ratio < 1/1.5), Q2 (1/1.5 < D7/D0 Ratio < 1/1.3), Q3 (1.3 < D7/D0 Ratio < 1.5) and Q4 (D7/D0 Ratio > 1.5)

The DESPs were divided into four quantitative categories according to their D7/D0 Ratio: Q1 (0 < D7/D0 Ratio < 1/1.5), Q2 (1/1.5 < D7/D0 Ratio < 1/1.3), Q3 (1.3 < D7/D0 Ratio < 1.5) and Q4 (D7/D0 Ratio > 1.5) (Fig. 5a). The quantifiable proteins from the four categories were plotted for GO enrichment-based cluster analysis. For the ‘biological process’ category, the significantly enriched GO terms were ‘small molecule biosynthesis process’, ‘organic acid biosynthetic process’, ‘primary metabolic process’, ‘organic substance metabolic process’, ‘macromolecule metabolic process’, ‘protein metabolic process’, ‘proteolysis’, ‘cellular macromolecule metabolic process’, ‘catabolic process’, ‘cellular protein metabolic process’ and ‘organic substance catabolic process’ (Fig.5b). For the ‘molecular function’ category, the significantly enriched GO terms were ‘calcium ion binding’, ‘metal ion binding’, ‘cation binding’, ‘carbohydrate binding’, ‘protein binding’, ‘phosphoglycerate kinase activity’, ‘kinase activity’ and ‘phosphotransferanse activity’ (Fig.5c).

The DESPs were enriched in five KEGG metabolic pathways, including ‘fructose and mannose metabolism’, ‘carbon fixation in photosynthetic organisms’, ‘protein processing in endoplasmic reticulum’, ‘biosynthesis of amino acids’ and ‘plant-pathogen interaction’ (Fig.5d).

Protein domain enrichment analysis revealed that the seven protein domains, including ‘ribosomal protein S5 domain 2’, ‘heat shock protein Hsp90’, ‘phosphoglycerate kinase, C-terminal’, ‘histidine kinase like ATPase’, ‘phosphoglycerate kinase, N-terminal’, ‘heat shock protein 70kD, peptide-binding domain’ and ‘heat shock protein 70kD, C-terminal domain’, were enriched in the DESPs (Fig. 5e and Additional file 8).

## Discussion

Chinese hickory is a popular nut-producing tree species with high economic value in China [9]. Grafting is one of the most frequently applied technique in asexual propagation of commercially grown Chinese hickory trees [34]. Grafting involves a number of physiological and biochemical processes and exact mechanism behind this technique yet to be explored in much detail [35]. Lysine succinylation is a widely identified PTM involved in diverse protein functions [16]. However, limited information on the changes in the succinylated proteins during the grafting process of Chinese hickory has been obtained.

In our study, the average numbers of succinylation sites in Chinese hickory and other reported organisms were counted (Fig. 1d). Data showed that the average succinylation sites in the reported organisms were more than that in Chinese hickory, indicating a low degree of succinylation in this nut plant. The role of highly diversified succinylation degrees in plants was needed to be addressed in the future.

Cytosol is the main area of cellular metabolism, whereas nucleus is the main repository of genetic information, and mitochondria is the main organelle required for the biosynthesis of succinyl-CoA and succinate [36–38]. In Chinese hickory, the succinylation was biased to occur in the cytosolic proteins, indicating a possible effect of succinylation on the activities of enzymes associated with cellular metabolism [17]. A great number of succinylated proteins were predicted to be mitochondria-localized proteins, suggesting that succinylation might play roles in maintaining and regulating metabolic function of Chinese hickory [39].

Many studies revealed that a large number of succinylated proteins are involved in various metabolic processes [28]. Several published succinyl-proteomes, including two mammals (*H. sapiens* and *M. musculus*), five microbes (*V. parahemolyticus*, *E. coli*, *C. glutamicum*, *M. tuberculosis*, *T. rubrum*), and six plants (*D. officinale*, *T. media*, *O. sativa*, *S. lycopersicum*, *C. sinensis* and *B. distachyon*) were extracted to evaluate the potential conservation of succinylation in the regulation of central metabolism [15, 18, 30, 40, 41]. The numbers of succinylation sites in the enzymes involved in glycolysis and TCA cycle were counted in the ten selected organisms (Fig. 4). Our data showed that the average number of succinylation sites in Chinese hickory was less than those in the ten representative organisms. Interestingly, the average number of succinylation sites in mammals and microbes was larger than those in plants, indicating a varied frequency of lysine succinylation among organisms.

In plants, such as grapevine, graft union formation affected the expression of the secondary metabolism-related genes [42]. In ‘Gold Finger’ grape berry, the contents of fructose, glucose and sucrose were affected by different rootstocks [43]. In our study, several fructose and mannose metabolism-related proteins, including fructose-bisphosphate aldolase-like protein, L-iditol 2-dehydrogenase and latex plastidic aldolase-like protein, were identified as succinylated proteins. These fructose and mannose metabolism-related proteins were observed to be significantly down-regulated during the grafting process of Chinese hickory, suggesting an important role of fructose and mannose in the grafting process (Fig. 5d). Systemic resistance in citrus was transmitted by grafting and mediated by mobile amino acids [44]. In the current study, eight amino acid biosynthesis-related proteins were identified as succinylated proteins, and most of them were significantly up-regulated during the grafting process (Fig. 5d), indicating that enhanced biosynthesis of amino acids might be required for a successful grafting of Chinese hickory.

HSPs exist ubiquitous in eukaryotes and increase in the expression of *HSP* genes were related with the enhanced resistance to environmental stresses in various plants [45, 46]. In total, seven HSPs with 11 succinylation sites were identified in Chinese hickory, including two HSP90s and five HSP70s (Additional file 8). Abscisic acid-induced HSP70 accumulation played a role in the heat tolerance of grafted cucumber plants [47]. Interestingly, all these HSPs were up-regulated during the grafting process of Chinese hickory, suggesting that over-accumulated HSPs might play a role in the tolerance of grafted Chinese hickory plants.

## Conclusions

In the current study, a comprehensive succinyl-proteome of Chinese hickory was presented. Quantitative analysis identified a number of differentially expressed succinylated proteins during the grafting process. Our data can act as important basic resource for the functional validation of succinylated proteins and a starting point for investigations into the molecular basis of lysine succinylation in the grafting process.

## Methods

### Plant samples and protein extraction

Chinese hickory (*Carya cathayensis* Sarg.) plants were planted in the plantation area at the campus of Zhejiang A&F University, Hangzhou, China. Plants were provided by the plantation (Zhejiang A&F University) where the formal identification of the plant material was undertaken by Dr. Daoliang Yan (Zhejiang A&F University). A voucher specimen of this material has not been deposited in a publicly available herbarium. All the plants were watered twice a week with a nutrient solution (1.425 mM NH_4_NO_3_, 0.323 mM NaH_2_PO_4_, 0.513 mM K_2_SO_4_, 0.998 mM CaCl_2_, 1.643 mM MgSO_4_, 0.009 mM MnCl_2_, 0.075 mM (NH_4_)_6_Mo_7_O_24_, 0.019 mM H_3_BO_3_, 0.155 mM CuSO_4_, 1 mM FeCl_3_, 0.070 mM citric acid and 0.152 mM ZnSO_4_). Plant samples were collected from the graft unions, including the cutting surfaces of the rootstocks (2 years old) and scions (1 year old), at 7 d after grafting. The samples from graft unions at the first day of grafting were used as the controls.

Plant samples were first ground with liquid N_2_. The cell powder was then transferred into a centrifuge tube (5 mL) and sonicated five times using a high intensity ultrasonic processor (Scientz, Ningbo, China) in pre-cooled lysis buffer containing urea (2 M), ethylenediamine tetraacetic acid (2 mM), dithiothreitol (10 mM) and Protease Inhibitor Cocktail VI (1%, MedChen Express, Monmouth Junction, USA). The remaining debris was discarded by centrifugation at 20,000 g at 4 °C for 10 min. The protein samples were precipitated with pre-cooled 15% TCA buffer for 2 h at − 20 °C. The supernatant was removed by centrifugation at 4 °C for 10 min. The remaining precipitates were washed with pre-cooled acetone for five times and then were re-dissolved in buffer containing 8 M urea and 100 mM TEAB (pH 8.0). The concentration of the final protein solution was determined by 2-D Quant kit according to manufacturer’s instructions (GE Healthcare, Little Chalfont, Buckinghamshire, UK) [48].

### Trypsin digestion and TMT labeling

The protein sample solution was reduced with 10 mM DTT for 1 h at 37 °C and alkylated with 20 mM IAA for 45 min at 25 °C in darkness. For trypsin digestion, the protein solution was diluted to the final concentration of less than 2 M by adding 100 mM triethylamine borane buffer. Trypsin was added at a trypsin:protein mass ratio of 1:50 for the first round overnight-digestion, followed by a second round 4 h-digestion at a trypsin:protein mass ratio of 1:100.

Then, sample peptides were desalted using a Strata X C18 SPE column (Phenomenex, Torrance, CA, USA). The resulting peptides were vacuum dried with centrifugation. Peptides were reconstituted in 0.5 M TEA buffer and processed by 6-plex TMT kit according to the manufacturer’s instructions (Thermo scientific, Shanghai, China). Briefly, one unit of TMT reagent, together with 100 μg of sample peptides, were mixed and reconstituted in acetonitrile solution. After incubation for 2 h at 25 °C, the resulting peptide mixture was desalted and vacuum-dried again.

### Affinity enrichment

To enrich Ksucc peptides, tryptic peptides dissolved in NETN buffer containing 100 mM NaCl, 1 mM EDTA, 50 mM Tris-HCl and 0.5% NP-40 (pH 8.0), were incubated with pre-washed antibody beads (PTM Biolabs, Hangzhou, China) at 4 °C overnight with gentle shaking. The beads were washed five times with NETN buffer and twice with ddH_2_O. The bound peptides were eluted from the beads with 0.1% trifluoroacetic acid. After combined and vacuum-dried by centrifugation, the eluted peptides were cleaned with C18 ZipTips (Millipore, Shanghai, China) according to its instructions before LC-MS/MS analysis.

### LC-MS/MS analysis

The LC-MS/MS analysis was carried out according to the procedure described previously [17]. Peptides were dissolved in 0.1% formic acid, directly loaded onto a reversed-phase pre-treated Acclaim PepMap 100 column (Thermo scientific, Shanghai, China). Peptide separation was carried out using a reversed-phase analytical Acclaim PepMap RSLC column (Thermo scientific, Shanghai, China). The resulting peptides were analyzed by Q Exactive™ plus hybrid quadrupole-Orbitrap MS (Thermo scientific, Shanghai, China).

The peptides were subjected to NSI source followed MS/MS in Q Exactive™ plus coupled online to the UPLC system (Thermo scientific, Shanghai, China). Intact peptides were detected in the Orbitrap at a high resolution of 70,000 and ion fragments were detected in the Orbitrap at a low resolution of 17,500. For MS scans, the m/z scan range was 350 to 1800, and the first mass was set as 100 m/z. The mass spectrometry proteomics data have already been deposited to the ProteomeXchange Consortium via the PRIDE partner repository with the dataset identifier PXD009584 (http://proteomecentral.proteomexchange.org/cgi/GetDataset?ID=PXD009584).

### Database search and protein annotation

The resulting MS/MS data was processed using MaxQuant integrated with Andromeda searching engine ver. 1.4.1.2. MS/MS were searched against *Carya* concatenated with reverse decoy database. Trypsin/P was used as cleavage enzyme allowing up to 4 missing cleavages, five modifications/peptide, and five charges. Mass errors were set to 10 ppm and 0.02 Da for precursor ions and fragment ions, respectively. The minimum number of peptides identified per protein was 1. Carbamido methylation on Cys was treated as fixed modification and succinylation on Lys was treated as variable modification. The false discovery rate (FDR) analysis was carried out according to the procedure described previously [17].

### Annotation and enrichment analysis

For Gene Ontology (GO) annotation, all succinylated proteins were searched against the UniProt-GOA database (http://www.ebi.ac.uk/GOA/). If a succinylated protein was not annotated by the UniProt-GOA database, InterProScan database (http://www.ebi.ac.uk/interpro/) was used for further annotation. For Kyoto Encyclopedia of Genes and Genomes (KEGG) pathway annotation, all succinylated proteins were searched the database using the online tool KEGG Automatic Annotation Server (https://www.genome.jp/tools/kaas/). The results were mapped to different KEGG pathways using the online service tool KEGG Mapper (https://www.genome.jp/kegg/mapper.html). For subcellular localization prediction, all succinylated proteins were searched using the software ‘wolfpsort’ (http://psort.hgc.jp/). Enrichment analyses of GO, KEGG and protein domain were carried out using a two-tailed Fisher’s exact test.

For hierarchical clustering, protein categories that were enriched in at least one of the clusters with a *P*-value < 0.05 were transformed by the x = −log10 (*P*-value) function. The *P*-value was transformed into Z-score after log transformation:

$$ Z\  sample-i=\frac{\log 2\left(\mathrm{Signal}\mathrm{s}\mathrm{ample}-\mathrm{i}\right)-\mathrm{Mean}\left(\mathrm{Log}2\left(\mathrm{Signal}\right)\mathrm{of}\ \mathrm{all}\ \mathrm{s}\mathrm{ample}\mathrm{s}\right)}{\mathrm{Standard}\ \mathrm{deviation}\ \left(\mathrm{Log}2\ \left(\mathrm{Signal}\right)\mathrm{of}\ \mathrm{all}\ \mathrm{s}\mathrm{ample}\mathrm{s}\right)} $$

These x-values were z-transformed and clustered by one-way hierarchical clustering (Euclidean distance, average linkage clustering) in Genesis and visualized by a heat map using the MeV software. The quantifiable proteins in this study were divided into four quantitative categories according to their D7/D0 Ratio: Q1 (0 < D7/D0 Ratio < 1/1.5), Q2 (1/1.5 < D7/D0 Ratio < 1/1.3), Q3 (1.3 < D7/D0 Ratio < 1.5) and Q4 (D7/D0 Ratio > 1.5). For the GO, KEGG and protein domain enrichment analyses, all of the proteins in each database were used as the background.

### Motif clustering analysis

Software Motif-X was used to analyze the amino acid model of sequences in specific positions of modity-21-mers (10 amino acids up- and down-stream of the succinylation sites). All the protein sequences in the database were used as background parameter. Firstly, enrichment analyses of all lysine succinylation sites were carried out using their significance *P* values. Categories were filtered out according to the criteria: *P* value < 0.05. Then, the *P* value matrices were calculated according to method reported in our previous study [17]. Heatmaps were applied to visualize the results using the ‘ggplots’ R-package (http://ggplot2.org/).

## Supplementary information

**Additional file 1: Figure S1.** A flow chart of the succinyl proteomics analysis.

**Additional file 2: Table S1.** The information of succinylated sites in each succinylated protein.

**Additional file 3: Figure S2.** The number of succinylated proteins in different species.

**Additional file 4: Table S2.** The number of succinylation sites on the enzymes engaged in the glycolysis of Chinese hickory.

**Additional file 5: Table S3.** The number of succinylation sites on the enzymes engaged in the TCA cycle of Chinese hickory.

**Additional file 6: Table S4.** A quantitative succinylome profiling of Chinese hickory during the grafting process.

**Additional file 7: Figure S3.** The numbers of differentially expressed succinylated proteins in Chinese hickory during the grafting process.

**Additional file 8: Table S5.** The differentially expressed HSPs.

## Data Availability

The datasets supporting the conclusions of this article are included within the article and its additional files. The mass spectrometry proteomics data have already been deposited to the ProteomeXchange Consortium via the PRIDE partner repository with the dataset identifier PXD009584 (http://proteomecentral.proteomexchange.org/cgi/GetDataset?ID=PXD009584). The data will be publicly accessible after 1th November, 2019.

## Abbreviations

ADH: Alcohol dehydrogenase
ALDH: Aldehyde dehydrogenase
ALDO: Fructose-bisphosphate aldolase
D: Aspartic acid
DESP: Differentially expressed succinylated protein
DLAT: Dihydrolipoyllysine-residue acetyltransferase
DLD: Dihydrolipoyl dehydrogenase
E: Glutamic acid
FBP: Fructose-1,6-bisphosphatase
FDR: False discovery rate
GAPDH: Glyceraldehyde-3-phosphate dehydrogenase
GO: Gene ontology
GTP: Phosphoeno/lpyruvate carboxykinase
HSP: Heat shock proteins
K: Lysine
KEGG: Kyoto Encyclopedia of Genes and Genomes
LC-MS/MS: High performance liquid chromatography-mass spectrometry
MDH: Malate dehydrogenase
OGDH: Oxoglutarate dehydrogenase
P: Proline
PGK: Phosphoglycerate kinase
PGM: Phosphoglucomutase
PTM: Post-translational modification
SUCLG: Succinate-CoA ligase
TMT: Tandem Mass Tag
TPI: Triosephosphate isomerase
