# Supplementary material for: Quantitative succinyl-proteome profiling of Chinese hickory (Carya cathayensis) during the grafting process
Source: BMC Plant Biol. 2019 Nov 4;19:467. doi: 10.1186/s12870-019-2072-8 (PMC6829946; doi:10.1186/s12870-019-2072-8)
Supplement: Supplementary file 1 — Additional file 1: Figure S1. A flow chart of the succinyl proteomics analysis. [file 12870_2019_2072_MOESM1_ESM.docx]

Figure S1 A flow chart of the succinyl proteomics analysis.
