# Supplementary material for: Quantitative succinyl-proteome profiling of Chinese hickory (Carya cathayensis) during the grafting process
Source: BMC Plant Biol. 2019 Nov 4;19:467. doi: 10.1186/s12870-019-2072-8 (PMC6829946; doi:10.1186/s12870-019-2072-8)
Supplement: Supplementary file 7 — Additional file 7: Figure S3. The numbers of differentially expressed succinylated proteins in Chinese hickory during the grafting process. [file 12870_2019_2072_MOESM7_ESM.docx]

Figure S3 The numbers of differential expressed succinylated proteins in hickory during grafting process.
